# Effect of extended hours dialysis on markers of chronic kidney disease-mineral and bone disorder in the ACTIVE Dialysis study

**DOI:** 10.1186/s12882-019-1438-3

**Published:** 2019-07-12

**Authors:** Zhipeng Zhan, Brendan Smyth, Nigel D. Toussaint, Nicholas A. Gray, Li Zuo, Janak R. de Zoysa, Christopher T. Chan, Chenggang Jin, Anish Scaria, Carmel M. Hawley, Vlado Perkovic, Meg J. Jardine, Ling Zhang

**Affiliations:** 1 Department of Nephrology, China-Japan Friendship Hospital, 2 Yinghuayuan E St, Chaoyang Qu, Beijing Shi, 100096 China; 2 Department of Nephrology, Second Clinical Medical Institution of North Sichuan Medical College, Nanchong, China; 3 The George Institute for Global Health, UNSW, 1 King St, Newtown, Sydney, 2042 Australia; 4 Sydney School of Public Health, University of Sydney, Sydney, Australia; 5 Department of Nephrology, The Royal Melbourne Hospital, Melbourne, Australia; 6 Sunshine Coast University Hospital, Birtinya, Australia; 7 Sunshine Coast Clinical School, University of Queensland, Birtinya, Australia; 8 Peking University People’s Hospital, Beijing, China; 9 North Shore Hospital, Auckland, New Zealand; 10 Department of Medicine, University of Auckland, Auckland, New Zealand; 11 University Health Network, Toronto, Canada; 12 School of Social Development and Public Policy, Beijing Normal University, Beijing, China; 13 Department of Nephrology, Princess Alexandra Hospital, Brisbane, Australia; 14 Renal Unit, Concord Repatriation General Hospital, Sydney, Australia

**Keywords:** Chronic haemodialysis, Mineral metabolism, Clinical trial, Dialysis dose, Phosphate

## Abstract

**Background:**

Chronic Kidney Disease - Mineral and Bone Disorder (CKD-MBD) is a significant cause of morbidity among haemodialysis patients and is associated with pathological changes in phosphate, calcium and parathyroid hormone (PTH). In the ACTIVE Dialysis study, extended hours dialysis reduced serum phosphate but did not cause important changes in PTH or serum calcium. This secondary analysis aimed to determine if changes in associated therapies may have influenced these findings and to identify differences between patient subgroups.

**Methods:**

The ACTIVE Dialysis study randomised 200 participants to extended hours haemodialysis (≥24 h/week) or conventional haemodialysis (≤18 h/week) for 12 months. Mean differences between treatment arms in serum phosphate, calcium and PTH; and among key subgroups (high vs. low baseline phosphate/PTH, region, time on dialysis, dialysis setting and frequency) were examined using mixed linear regression.

**Results:**

Phosphate binder use was reduced with extended hours (− 0.83 tablets per day [95% CI -1.61, − 0.04; *p* = 0.04]), but no differences in type of phosphate binder, use of vitamin D, dose of cinacalcet or dialysate calcium were observed. In adjusted analysis, extended hours were associated with lower phosphate (− 0.219 mmol/L [− 0.314, − 0.124; *P* < 0.001]), higher calcium (0.046 mmol/L [0.007, 0.086; *P* = 0.021]) and no change in PTH (0.025 pmol/L [− 0.107, 0.157; *P* = 0.713]). The reduction in phosphate with extended hours was greater in those with higher baseline PTH and dialysing at home.

**Conclusion:**

Extended hours haemodialysis independently reduced serum phosphate levels with minimal change in serum calcium and PTH levels. With a few exceptions, these results were consistent across patient subgroups.

**Trial registration:**

Clinicaltrials.gov NCT00649298. Registered 1 April 2008.

**Electronic supplementary material:**

The online version of this article (10.1186/s12882-019-1438-3) contains supplementary material, which is available to authorized users.

## Background

Abnormalities of mineral and bone metabolism are common in patients with chronic kidney disease (CKD) and contribute significantly to higher rates of mortality and morbidity including cardiovascular disease and fracture [1–5]. Reduced renal function is associated with impaired phosphate excretion and diminished conversion of 25-hydroxyvitamin D to 1,25-dihydroxyvitamin D resulting in hypersecretion of parathyroid hormone (PTH) and the osteocyte-derived phosphatonin, fibroblast growth factor-23 (FGF-23). These changes lead to dysregulation of bone turnover and gastrointestinal absorption of calcium and phosphate, and tissue inhibition of calcification with consequences for bone integrity, mineral metabolism and vascular calcification [6]. In recognition of these intimate associations, the term ‘Chronic Kidney Disease-Mineral and Bone Disorder’ (CKD-MBD) was defined to encompass the disturbances of mineral metabolism, renal bone disease and vascular calcification, together with patient-level outcomes of fracture, cardiovascular disease and mortality in patients with CKD [7].

Standard treatment for CKD-MBD, including phosphate binders, vitamin D analogues and parathyroidectomy, is complex and not only lacks a firm evidenced-based foundation for efficacy, but also has the potential for harm [8, 9]. Hyperphosphataemia is the focus of considerable clinical attention, managed by dietary restriction, phosphate binders and dialysis manipulation. Phosphate is predominantly intracellular and moves slowly between the intracellular and extracellular spaces. As such, conventional thrice-weekly haemodialysis is frequently insufficient to attain negative phosphate balance and fewer than half of dialysis patients achieve values recommended by several clinical practice guidelines [10]. Aggressive adherence to a low-phosphate diet can potentially lead to low dietary protein intake and malnutrition [11] and the large number of phosphate binder tablets needed to control hyperphosphataemia in end-stage kidney disease (ESKD) can result in pill burden, increased disease intrusion, abdominal symptoms and potentially impact the absorption of other medications [12]. Frequent haemodialysis (5–6 times per week) has been shown to reduce serum phosphate in a number of small randomised trials [13–15]. Comparisons of 4 h vs. 8 h dialysis sessions confirm greater phosphate removal [16, 17] and observational research has associated each increase of 30 min of dialysis session time with a decrease of 0.013–0.05 mmol/L phosphate [18]. Yet, the longer term effects of extended dialysis hours achieved predominantly via longer sessions on key markers of CKD-MBD remains less well understood.

The ACTIVE Dialysis trial randomised 200 participants to extended hours dialysis (≥ 24 h per week) or conventional hours dialysis (≤ 18 h per week) and found no impact on the primary outcome of change in quality of life as measured by EuroQOL-5 Dimensions (EQ-5D) [19]. Extended hours dialysis was, however, associated with a reduction in serum phosphate, a small increase in serum calcium, no change in PTH and a decrease in phosphate binder tablet burden. These results imply a response by the treating physician to the study intervention, thus raising the possibility that the true impact of the intervention on individual markers of CKD-MBD may have been obscured by secondary changes in associated therapies. Furthermore, no study has examined the potential impact of more intensive dialysis on CKD-MBD markers among patient subgroups. The present analyses were undertaken to assess the impact of extended hours dialysis on CKD-MBD markers in pre-specified patient subgroups and accounting for concurrent changes in non-dialytic CKD-MBD therapies.

## Methods

### Study design

The ACTIVE Dialysis trial was an international, multicentre, open-label blinded endpoint-assessment trial randomising participants to conventional (≤ 18 h per week) or extended hours (≥ 24 h per week) haemodialysis with a minimum of three sessions per week for 12 months. The primary outcome was change in quality of life from baseline assessed by EQ-5D and secondary outcomes included medication usage and laboratory values. The design and baseline characteristics have been previously reported in detail [20]. A central web-based interface was used to randomise participants in a 1:1 ratio stratified according to geographical region (Australia/New Zealand vs China/Canada), dialysis setting (institution vs home), and dialysis duration at study entry (≤ 6 months vs > 6 months). Blinding of participants and treating clinicians was not feasible, however endpoint-assessment was conducted in a blinded fashion. The allocated treatment was commenced within 1 month of randomisation and continued for 12 months unless participants died, withdrew consent or were transferred to a setting that could not offer both therapies.

### Data collection

Participants were assessed at baseline and 3-monthly intervals. Data collection is described in detail elsewhere [20], but included serum phosphate, PTH and calcium (corrected for albumin). Prescribed medications were recorded including the number and type of phosphate binders, use of vitamin D analogues and dose of cinacalcet. Dialysate composition including dialysate calcium concentration and the addition of phosphate salts was also recorded. Dietary phosphate intake was not recorded. Adverse events including fractures and parathyroidectomy were recorded.

### Outcomes

The current study examined three key CKD-MBD parameters: serum phosphate, calcium (corrected for albumin) and PTH.

#### Primary outcomes

The primary outcome was the mean difference in each parameter between the extended and standard dialysis arms, adjusted for confounding participant characteristics and for changes in associated non-dialytic therapies: total number of phosphate binders, use of calcitriol/alfacalcidol, dose of cinacalcet and dialysate calcium.

#### Main secondary outcomes

The main secondary outcomes were the interactions between subgroups derived from six pre-defined criteria and the unadjusted mean difference in parameters between treatment arms. The criteria used to define subgroups included two biochemical markers of CKD-MBD: high vs. low baseline phosphate and high vs. low PTH (both defined by the median value); one dialysis-delivery parameter: dialysis session frequency (≤ 3 vs. > 3 per week); and three pre-defined criteria used in the main ACTIVE study analysis: dialysis location (home vs. institution), dialysis vintage (≤ 6 months vs. > 6 months) and global region (China vs. Australia/New Zealand/Canada).

#### Other secondary outcomes

Change in associated non-dialytic CKD-MBD therapies (as described above) was compared between treatment groups, along with the incidence of fractures and parathyroidectomy. In addition, the likelihood of achieving optimal levels of CKD-MBD parameters was explored with reference to international guidelines [10] and the normal population limits from the lead site in China: 1.13–1.78 mmol/L for phosphate, 2.10–2.50 mmol/L for calcium and 2 to 9 times the local laboratory upper limit of normal for PTH.

### Statistical analysis

Data from each of the four 3-monthly study visits was used in the analysis and treatment allocation to extended or standard hours dialysis was defined by intention-to-treat. Normally distributed variables were summarised as mean and standard deviation and non-normally distributed variables as median and interquartile range. The primary outcome of mean difference in parameters by treatment allocation was estimated using mixed linear regression adjusted for follow-up time point, baseline parameter, age, gender, dialysis location, dialysis vintage, global region and baseline and follow-up visit non-dialytic therapy characteristics (as specified above). The main secondary outcomes were defined by mixed linear regression of each parameter adjusted for baseline parameter, follow up time point and treatment allocation with an interaction with the relevant subgroup criteria. The relationship between treatment allocation and achievement of optimal CKD-MBD parameters was estimated by mixed logistic regression adjusted for follow up time point. The mean difference in number of phosphate binders, dialysate calcium and cinacalcet dose (restricted to those participants who received cinacalcet at ≥1 visit) was described by mixed linear regression adjusted by treatment allocation, baseline value and follow up time point. All models included a random intercept per participant and linear models also included a first-order autoregressive correlation structure to account for the correlation between values from sequential follow up visits. PTH was log_10_ transformed prior to analysis due to a highly skewed distribution. Proportion of participants receiving vitamin D analogues and calcium- or non-calcium-based phosphate binders in each group at each follow up time point was analysed by chi-squared test. All *p*-values were two-sided. No adjustment for multiple testing was made and missing values were not imputed. Analysis was conducted using Stata 15 (StataCorp, USA).

### Ethical principles

The ACTIVE Dialysis study was carried out in accordance with the principles of the Helsinki Declaration and approval was obtained from Human Research Ethics Committee of Northern Sydney Central Coast Health, NSW, Australia (HREC/09/HARBR/26). Approval was also obtained from each participating site according to their local practice. Written, informed consent was obtained from all participants. The study was registered at clinicaltrials.gov (NCT00649298).

## Results

### Baseline results, Dialysis therapy and CKD-MBD events

Two hundred participants were recruited from four countries - China (62.0%), Australia (29.0%), Canada (5.5%) and New Zealand (3.5%). The concentrations of serum phosphate, corrected calcium and intact PTH were similar between groups (Table 1). Median total weekly dialysis hours during the study period was 12 (IQR 12–16) and 24 (24-24) in the standard and extended arms, respectively. Use of haemodiafiltration (HDF) was more common during the study period among the standard arm than the extended arm (22.2% vs. 14.2% of sessions), although this did not reach significance (odds ratio [OR] for HDF 0.32 [95%CI 0.01, 1.02]; *P* = 0.056). There were no significant differences in dialysate concentrations of sodium, potassium or calcium. Blood flow rates were lower in the extended arm during the study period (250 mL/min [IQR 230–300] vs. 280 mL/min [250–300]; mean difference 23 mL/min [95% CI 11, 34]; *P* < 0.001). Dialysate flow rate was 500 mL/min at 90.6% of study visits and median flow rates did not differ (500 mL/min [IQR 500–500]), although a small number of outlying values resulted in mean dialysate flow rates being lower in the extended arm (mean difference 25 mL/min [95%CI 9, 42]; *P* = 0.003) (Additional file 1). Two fractures and three parathyroidectomies were recorded in five participants during the study period (Standard arm: one fracture, two parathyroidectomies; extended arm: one fracture and one parathyroidectomy).

**Table 1:** Baseline participant characteristics

Characteristics	Standard (*n* = 100)	Extended (*n* = 100)	Total (*n* = 200)	*P*-value
Mean age at randomization, yr. (SD)	51.6 (11.5)	52.1 (12.7)	51.8 (12.1)	0.79
Male, n (%)	70 (70.0)	69 (69.0)	139 (69.5)	0.88
Country, n (%)				
Australia	28 (28.0)	30 (30.0)	58 (29.0)	0.96
Canada	6 (6.0)	5 (5.0)	11 (5.5)	
China	62 (62.0)	62 (62.0)	124 (62.0)	
New Zealand	4 (4.0)	3 (3.0)	7 (3.5)	
Body Mass Index (kg/m^2^)	26.7 (6.1)	25.5 (6.4)	26.1 (6.2)	0.16
Number of dialysis sessions per wk., n (%)				
2	1 (1.0)	1 (1.0)	2 (1.0)	0.30
3	82 (82.0)	86 (86.0)	168 (84.0)	
≥ 4	17 (17.0)	13(13.0)	30 (15.0)	
Total number of hr. on dialysis per wk				
Median (IQR)	12.4 (12.0–16.0)	12.0 (12.0–15.0)	12.0 (12.0–15.0)	0.10
Duration on dialysis at enrolment, median (IQR) in yr.	2.63 (0.97–6.74)	2.43 (0.67–5.04)	2.48 (0.72–6.00)	0.37
Dialysis site at enrolment, n (%)				
Home	12 (12.0)	11 (11.0)	23 (11.5)	0.83
In-centre/satellite	88 (88.0)	89 (89.0)	177 (88.5)	
Intended dialysis site for study treatment, n (%)				
Home	25 (25)	26 (26)	51 (25.5)	0.87
In-centre/satellite	75 (75.0)	74 (74.0)	149 (74.5)	
Laboratory parameters, mean (SD)				
Phosphate (mmol/L)	1.77 (0.59)	1.86 (0.53)	1.82 (0.56)	0.31
PTH (pmol/L) median (IQR)	20.7 (10.9–43.5)	28.5 (11.7–46.2)	24.0 (11.3–46.0)	0.33
Corrected Calcium (mmol/L)	2.28 (0.24)	2.27 (0.20)	2.28 (0.22)	0.72
Albumin (g/L)	39.0 (4.5)	39.0 (4.7)	39.0 (4.6)	0.96
Phosphate binder use, n (%)				
Calcium-based	57 (57)	61 (61)	118 (59)	0.28
Non-calcium based	11 (11)	7 (7)	18 (9)	
Both non-calcium and calcium based	12 (12)	6 (6)	18 (9)	
No binder	20 (20)	26 (26)	46 (23)	
Phosphate binder burden (pills/day), median (IQR)	3.5 (1.5–6)	3 (0–6)	3 (1–6)	0.21
Cinacalcet use, n (%)	4 (4.1)	7 (7.0)	11 (5.6)	0.37
Calcitriol and analogues, n (%)				
No vitamin D	45 (46)	48 (48)	93 (47.0)	0.37
Calcitriol/Alfacalcidiol	47 (48)	49 (49)	96 (48.5)	
Cholecalciferol	6 (6)	2 (2)	8 (4.0)	
Both	0 (0)	1 (1)	1 (0.5)	
Dialysate calcium (mmol/L), n (%)				
Low (≤1.3)	36 (36.0)	32 (32.0)	68 (34.0)	0.63
Mid (1.5)	58 (58.0)	64 (64.0)	122 (61.0)	
High (≥1.6)	6 (6.0)	4 (4.0)	10 (5.0)	

### Unadjusted change in phosphate, PTH and calcium with extended hours dialysis

**Fig. 1:**
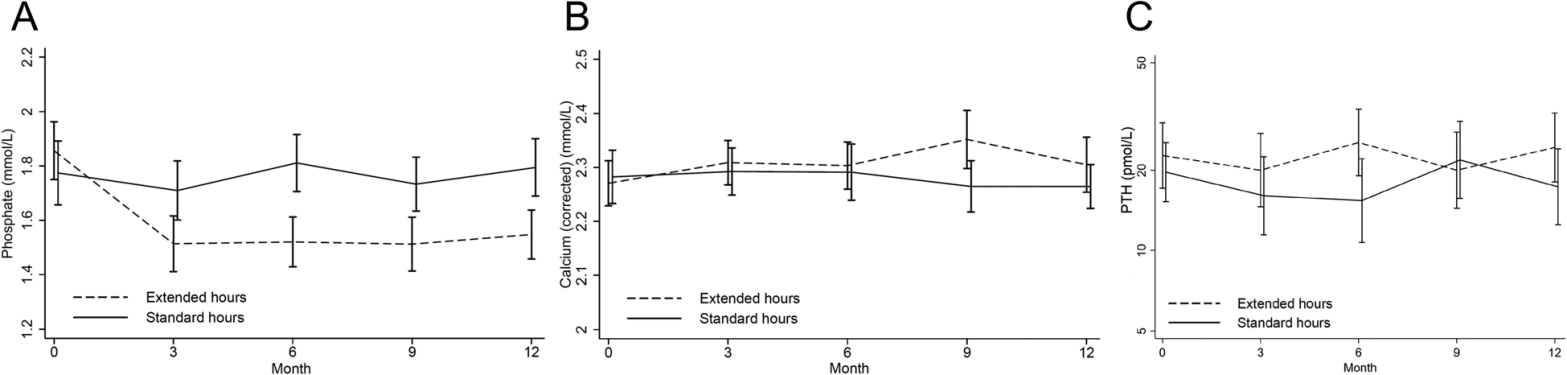
Mean (95% CI) serum phosphate, calcium and PTH levels in each group over the 12-month study period. **a**. Phosphate **b**. Calcium (corrected for albumin) **c**. PTH

Over the 12 months of follow up, serum phosphate was lower in the extended hours group (mean difference: − 0.25 mmol/L [− 0.30, − 0.15, *p* < 0.0001]). This effect was apparent from 3 months and maintained over the duration of the study (Fig. 1). There was a small, but statistically significant increase in corrected calcium of 0.05 mmol/L (mean difference: 0.01, 0.09, *p* = 0.013) and no significant change in PTH. The lack of effect on PTH was supported by a *post-hoc* sensitivity analysis restricted to the 143 participants who were never exposed to cinacalcet.

### Change in non-dialytic CKD-MBD therapies

**Fig. 2:**
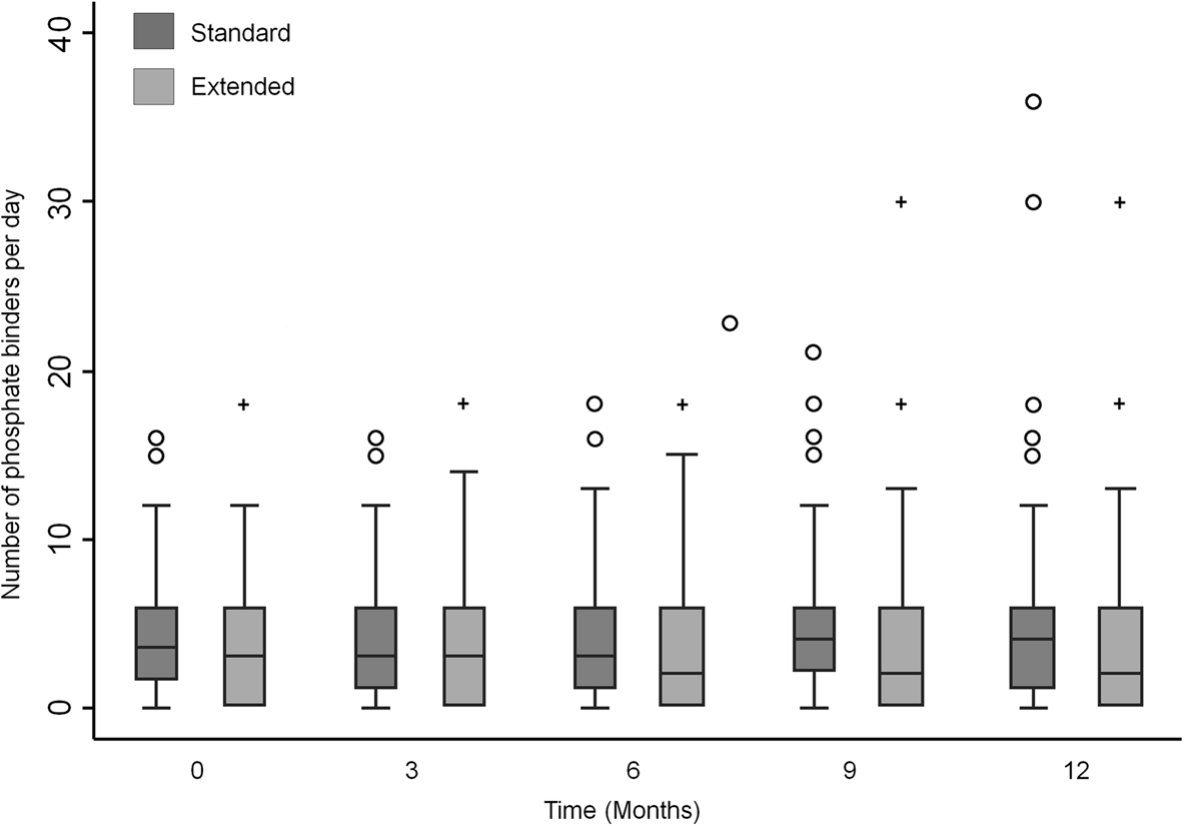
Median and interquartile range of the total number of phosphate binder tablets in each group over the 12-month study period

There was a significant reduction in the mean daily number of phosphate binders of − 0.83 tablets per day (− 1.63, − 0.03, *p* = 0.04) (Fig. 2). Among participants on phosphate binders, there was no change in the proportion on calcium-based vs. non-calcium based binders. There were no differences in the proportion of participants using vitamin D derivatives (Table 2) or in dialysate calcium (mean difference 0.003 mmol/L [95% CI -0.021, 0.027], *p* = 0.79). Among the twenty-three participants receiving cinacalcet on at least one study visit, no difference in mean daily dose was observed during the follow up period (− 11.7 mg [95% CI -29.0, 5.9]; *p* = 0.19) (Table 3). In sites outside China, an average of 11.4% of participants in the extended arm required supplemental phosphate (either in the dialysate or per-oral) as opposed to 0.8% in the standard arm. At sites in China, dialysate supplementation with phosphate was not performed and dietary phosphate supplementation was not included in data collection. As such, phosphate supplementation was not considered in further analysis.

**Table 2:** Proportion of patients on active vitamin D (calcitriol/alfacalcidol) or cholecalciferol by visit and treatment

	Baseline		Month 3		Month 6		Month 9		Month 12	
	Standard	Extended	Standard	Extended	Standard	Extended	Standard	Extended	Standard	Extended
No vitamin D	45.9	48	40.2	51.6	45.3	47.8	44.1	44.3	48.9	47.1
Active vitamin D	48	49	53.6	45.4	47.4	48.9	48.4	50	41.3	47.1
Cholecalciferol	6.1	2	4.1	3.1	6.3	3.3	7.5	5.7	8.7	4.6
Both	0	1	2.1	0	1.1	0	0	0	1.1	1.2
*P*-value	0.37		0.24		0.6		0.88		0.68

**Table 3:** Mean dose and usage of cinacalcet Twenty-three participants were receiving cinacalcet on at least one study visit. Mean dose includes only those participants receiving cinacalcet at that visit. Overall mean difference includes all 23 participants and all visits

	Standard		Extended		Between-Group difference	
	N/11	Mean dose (SD)	N/12	Mean (sd)	Mean difference (95% CI)	*P*-value
Baseline	4	50.0 (14.1)	7	51.4 (56.7)	1.4 (−65.2, 68.1)	
3-month	8	43.1 (15.8)	7	34.3 (11.3)	−8.8 (−24.4, 6.7)	
6-month	9	41.7 (15.4)	6	35.0 (12.3)	−6.7 (−22.9, 9.6)	
9-month	10	42.5 (14.8)	6	48.3 (36.0)	5.8 (−21.4, 33.0)	
12-month	8	46.9 (15.8)	7	30.3 (11.0)	−16.6 (−32.0, −1.2)	
Overall mean difference					− 11.7 (−29.0, 5.9)	0.19

### Multivariable analysis of change in laboratory parameters

The treatment effect of extended hours dialysis on serum phosphate and calcium remained in multivariable analysis (*p* < 0.001 and *p* = 0.021, respectively) (Tables 4 and 6). Other independent predictors of lower serum phosphate levels were more than 3 dialysis sessions per week (*p* = 0.008); being in Australia, New Zealand or Canada (vs. China) (*p* = 0.017); dialysing at an institution (vs. home) (*p* = 0.037) and having a lower serum phosphate at baseline (p < 0.001). Other independent predictors of higher serum calcium were use of active vitamin D (*p* = 0.041) and higher baseline serum calcium (*p* < 0.001). Extended hours dialysis was not associated with change in PTH in the multivariable model (*p* = 0.713) (Table 5). Lower PTH levels were associated with higher baseline dialysate calcium (*p* = 0.043), older age (*p* = 0.01) and being in Australia, New Zealand or Canada (vs. China) (*p* = 0.003).

**Table 4:** Adjusted model of change in phosphate (mmol/L), calcium (mmol/L) and PTH (log10, pmol/L)

	Phosphate		Calcium		PTH (log10)	
Variable	Beta (95% CI)	*P*-value	Beta (95% CI)	*P*-value	Beta (95% CI)	*P*-value
Extended hours dialysis (ref = Standard)	−0.219 (−0.314, − 0.124)	< 0.001	0.046 (0.007, 0.086)	0.021	0.025 (− 0.107, 0.157)	0.713
Baseline parameter (phosphate/calcium/PTH)	0.267 (0.164, 0.369)	< 0.001	0.576 (0.428, 0.724)	< 0.001	0.523 (0.382, 0.663)	< 0.001
Use of active vitamin D (ref = No use)	0.067 (−0.017, 0.152)	0.119	0.048 (0.002, 0.094)	0.041	−0.03 (− 0.165, 0.104)	0.661
Baseline use of active vitamin D	−0.046 (− 0.155, 0.064)	0.415	− 0.044 (− 0.096, 0.008)	0.098	0.049 (− 0.105, 0.202)	0.533
Dose of cinacalcet	0.002 (− 0.001, 0.006)	0.23	< 0.001 (− 0.002, 0.001)	0.806	0.002 (− 0.004, 0.007)	0.54
Baseline dose of cinacalcet	−0.001 (− 0.003, 0.002)	0.593	0.001 (− 0.001, 0.003)	0.2	< 0.001 (− 0.005, 0.005)	0.876
Dialysate calcium	− 0.338 (− 0.748, 0.072)	0.107	− 0.1 (− 0.271, 0.071)	0.253	0.043 (− 0.517, 0.603)	0.88
Baseline dialysate calcium	0.32 (− 0.067, 0.708)	0.105	0.116 (− 0.094, 0.326)	0.277	−0.734 (−1.445, − 0.023)	0.043
Number of phosphate binders	< 0.001 (− 0.011, 0.01)	0.965	0.002 (− 0.003, 0.006)	0.503	− 0.008 (− 0.017, 0.001)	0.072
Baseline number of phosphate binders	0.007 (− 0.009, 0.023)	0.393	− 0.001 (− 0.01, 0.007)	0.759	−0.004 (− 0.032, 0.024)	0.797
Age	−0.002 (− 0.006, 0.002)	0.278	< 0.001 (− 0.002, 0.001)	0.605	−0.007 (− 0.013, − 0.002)	0.01
Female (ref = Male)	−0.018 (− 0.125, 0.09)	0.749	−0.023 (− 0.064, 0.019)	0.284	0.02 (− 0.11, 0.15)	0.76
More than 3x weekly dialysis (ref = 3x or fewer)	−0.113 (− 0.196, − 0.03)	0.008	0.029 (− 0.008, 0.067)	0.128	−0.024 (− 0.137, 0.09)	0.68
Non-China (ref = China)	−0.18 (− 0.328, − 0.032)	0.017	0.004 (− 0.056, 0.064)	0.89	−0.267 (− 0.445, − 0.089)	0.003
Home dialysis (ref = Institution)	0.183 (0.011, 0.356)	0.037	−0.019 (− 0.079, 0.04)	0.527	0.08 (− 0.157, 0.318)	0.509
< 6 months on dialysis (ref= > 6 months)	−0.062 (− 0.201, 0.077)	0.381	0.012 (− 0.031, 0.055)	0.592	−0.166 (− 0.342, 0.011)	0.066

### CKD-MBD treatment target achievement

Achievement of serum phosphate levels within the target range over the duration of the study was more common in the extended hours dialysis group (relative risk [RR] 1.21 [1.04, 1.43]; *p* = 0.016). There were no differences between groups in the proportion of patients achieving target ranges for serum calcium (RR 1.03 [0.93, 1.14]; *p* = 0.61) and PTH (RR 1.09 [0.89, 1.34]; *p* = 0.40).

### Subgroup analysis of change in laboratory parameters

**Fig. 3:**
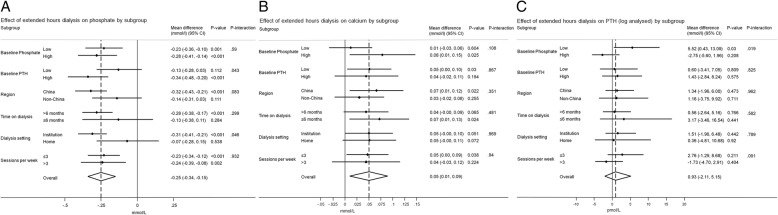
Mean (95% CI) between-group difference in laboratory parameters with extended hours dialysis by subgroups. **a**. Phosphate. **b**. Calcium. **c**. PTH

The impact of extended hours dialysis on serum phosphate, calcium and PTH was generally consistent across tested subgroups (Fig. 3). The exceptions to this were that the effect of treatment allocation on phosphate interacted significantly with both baseline level of PTH (p for interaction = 0.043) and dialysis location (p for interaction = 0.046); such that those with high baseline PTH and dialyzing at an institution experienced a greater reduction in serum phosphate with extended hours dialysis. In addition, there was a significant interaction between the effect of treatment allocation on PTH and baseline phosphate (p for interaction = 0.019); such that those with a low baseline serum phosphate experienced a small increase in PTH if assigned to extended hours dialysis.

## Discussion

These results demonstrate that the effect of extended hours dialysis on key markers of CKD-MBD (lower phosphate, higher calcium and no change in PTH) is independent of changes to associated therapies. With few exceptions, these effects were consistent across a range of important patient subgroups. Moreover, patients on extended hours dialysis were more likely to achieve strict therapeutic targets and to experience a reduction in the need for oral phosphate binders.

Elevated serum phosphate is a risk factor for cardiovascular death in patients on dialysis, leading to the hypothesis that phosphate-lowering therapy may improve survival, although RCT (randomised controlled trial) evidence is lacking [21, 22]. Our study confirms that extended hours haemodialysis can reduce serum phosphate levels, leading to a significant difference compared to conventional dialysis as early as 3 months. The results of our study are consistent with data from the three randomised trials of frequent dialysis. The Frequent Hemodialysis Network (FHN) trials demonstrated that intensive dialysis, delivered as short daily [13] or long nocturnal schedules [15] (each 5-6x per week), reduced serum phosphate levels by 0.19 mmol/L and 0.52 mmol/L respectively, relative to standard dialysis of 3 sessions per week (< 5 h each). In the FHN Daily trial, intensive haemodialysis significantly lowered the daily dose of phosphate binders, while in the FHN Nocturnal trial it led to binder discontinuation in 75% of patients [23]. An earlier study of frequent nocturnal dialysis demonstrated similar results [14]. With the exception of the latter study, in which median PTH in the frequent dialysis group was lower than the standard dialysis group by 10.5 pmol/L (*P* = 0.05) at study end, intensive dialysis appeared to have no meaningful effects on serum calcium and PTH concentrations. This is consistent with the present study, in which no meaningful change in either parameter was noted.

Phosphate clearance on haemodialysis is complex. It is a predominantly intracellular solute and movement between the intravascular space and the main reservoir of body phosphate stores is relatively slow. Phosphate clearance is highest at the start of dialysis and falls steadily over the first 1–2 h of a session before reaching a plateau as the rate of mobilisation of intracellular phosphate equals the ongoing dialytic clearance [24]. While frequent dialysis maximises the time spent in the early part of a dialysis session when phosphate clearance is highest, the present study confirms that the continued clearance (albeit at a lower rate) that occurs with extending dialysis time is also an effective means of controlling serum phosphate.

Although the optimal serum phosphate levels in patients on dialysis are unknown, both extremes of high or low serum phosphate levels are associated with an increased risk of mortality in dialysis patients [25]. The Kidney Disease Improving Global Outcomes (KDIGO) CKD-MBD guidelines suggest lowering serum phosphate towards the normal range [10]. In the present study, extended hours haemodialysis increased the proportion of patients achieving a normal serum phosphate compared with patients on conventional dialysis. Moreover, those patients may have liberalised their dietary phosphate intake, which may in turn ensure adequate protein intake and help to avoid malnutrition. Low body weight and poor nutrition are also strong predictors of adverse outcome in dialysis patients and the ability to avoid dietary restrictions is hypothesised to contribute to the increased survival seen in prospective cohort studies of more frequent or extended hours dialysis [26]. Beyond this, a reduction in dietary restrictions could avoid the stress and anxiety that commonly accompany diet in patients on dialysis [27]. Similarly, a reduction in phosphate binder requirement may minimise the high pill burden and common gastrointestinal side effects of these medications, known to contribute to poor patient compliance and lower quality of life [12]. Finally, we found that the proportion of non-calcium phosphate binders was reduced, although with no difference in calcium-containing binders. One potential hypothesis for this finding is that clinicians may preferentially cease the more expensive non-calcium phosphate binders initially when serum phosphate has improved, especially in countries where more costly phosphate binders are not financially reimbursed.

In subgroup analyses, the statistical interaction between the effect of extended hours dialysis on serum phosphate and dialysis site indicates that those dialysing at an institution (as opposed to at home) may derive greater benefit on their phosphate from longer dialysis hours. This may reflect differences in age, comorbidities and adherence to medication and dietary prescriptions. However, it is also possible that home patients over-reported the actual amount of dialysis that they performed, resulting in a relatively higher delivered dose of dialysis in the institution patients. There was also an interaction between the effect on serum phosphate and baseline PTH, with those having a high baseline PTH experiencing a greater reduction in serum phosphate with extended hours dialysis. The cause of this is unclear. High PTH states result in an expanded pool of exchangeable bone calcium [28], whether this is accompanied by an increase in exchangeable phosphate such that equilibration between bone and serum phosphate is more rapid and thus facilitating greater phosphate clearance is not known. There was also an interaction between effect on PTH and baseline phosphate, raising the hypothesis that extended hours dialysis is associated with an increase in PTH in those with low baseline phosphate. RCT with larger subject numbers are needed to verify this speculation. We also found a significant increase in serum calcium by 0.05 mmol/L in those on extended hours haemodialysis, although the clinical relevance of this is uncertain. This change remained after adjustment for changes in phosphate, PTH, dialysate calcium, use of active vitamin D and cinacalcet and there were no between-group differences in use of calcium-based phosphate-binding agents. Previous frequent haemodialysis trials have not seen this effect [13–15]. Future studies of extended hours dialysis should report this outcome.

The strengths of this study include a clear separation in dialysis times between the two groups that was maintained over the duration of the study [19]. The inclusion of a broad patient population also provides greater generalisability. However, despite being the largest randomised trial of extended hours dialysis, the cohort is still relatively small. This limits the power of the study to detect subgroup differences and the results of this present secondary analysis remain exploratory. Along with the short duration of the study, this also prohibited any investigation of clinical endpoints (such as fracture or cardiovascular disease). A further limitation was the lack of serum levels of calcidiol (25-hydroxyvitamin D). While unlikely to be greatly affected by increased dialysis hours (it is predominantly protein-bound and has a high volume of distribution) [29], we cannot determine if unobserved changes in calcidiol levels influenced the behaviour of other markers of CKD-MBD. Other limitations include the lack of comprehensive data on changes in dietary and dialysis phosphate, dialysate magnesium and bicarbonate, and the absence of more specific serum markers of bone turnover or radiographic investigation of vascular calcification.

## Conclusions

The improvement in serum phosphate associated with extended hours haemodialysis was independent of changes in other CKD-MBD therapies and was consistent across a range of important patient subgroups. The observed differences in the impact of extended hours dialysis on phosphate seen in those with high baseline PTH or dialysing in an institution, or on PTH in those with low phosphate require confirmation in larger studies.

## Additional file

Additional file 1 : **Table S1.** Additional baseline characteristics. **Table S2.** Dialysate composition, flow rates and use of HDF over the duration of the study. **Table S3.** Serum parameters and dialysis adequacy over the duration of the study. **Table S4.** Blood pressure and fluid status changes over the duration of the study. **Figure S1.** Dialysate calcium over the duration of the study. (DOCX 264 kb)

## Data Availability

The datasets analysed during the current study are available from the corresponding author on reasonable request. Interested parties are referred to the main ACTIVE Dialysis publication: Jardine MJ, Zuo L, Gray NA, de Zoysa JR, Chan CT, Gallagher MP, et al. A Trial of Extending Hemodialysis Hours and Quality of Life. Journal of the American Society of Nephrology. 2017;28(6):1898–911.

## Abbreviations

ACTIVE Dialysis: A Clinical Trial of IntensiVE Dialysis
CKD: Chronic kidney disease
CKD-MBD: Chronic kidney disease – mineral and bone disorder
EQ 5D: EuroQOL-5 dimensions
ESKD: End-stage kidney disease
FGF-23: Fibroblast growth factor-23
FHN: Frequent Hemodialysis Network
KDIGO: Kidney Disease Improving Global Outcomes
PTH: Parathyroid hormone

## References

[1] Kovesdy CP, Ahmadzadeh S, Anderson JE, Kalantar-Zadeh K (2008). Secondary hyperparathyroidism is associated with higher mortality in men with moderate to severe chronic kidney disease. Kidney Int.

[2] Weiner DE, Tighiouart H, Amin MG, Stark PC, MacLeod B, Griffith JL (2004). Chronic kidney disease as a risk factor for cardiovascular disease and all-cause mortality: a pooled analysis of community-based studies. J Am Soc Nephrol.

[3] Foley RN, Parfrey PS, Sarnak MJ (1998). Clinical epidemiology of cardiovascular disease in chronic renal disease. Am J Kidney Dis.

[4] Muntner P, He J, Astor BC, Folsom AR, Coresh J (2005). Traditional and nontraditional risk factors predict coronary heart disease in chronic kidney disease: results from the atherosclerosis risk in communities study. J Am Soc Nephrol.

[5] Eddington H, Hoefield R, Sinha S, Chrysochou C, Lane B, Foley RN (2010). Serum phosphate and mortality in patients with chronic kidney disease. Clin J Am Soc Nephrol.

[6] Silver J, Naveh-Many T (2013). FGF-23 and secondary hyperparathyroidism in chronic kidney disease. Nat Rev Nephrol.

[7] Moe SM, Drueke T, Lameire N, Eknoyan G (2007). Chronic kidney disease-mineral-bone disorder: a new paradigm. Adv Chronic Kidney Dis.

[8] Goldsmith D, Covic A, Vervloet M, Cozzolino M, Nistor I, Chronic Kidney Disease-Mineral Bone Disease working g (2015). Should patients with CKD stage 5D and biochemical evidence of secondary hyperparathyroidism be prescribed calcimimetic therapy? An ERA-EDTA position statement. Nephrol Dial Transplant.

[9] Kalantar-Zadeh K, Shah A, Duong U, Hechter RC, Dukkipati R, Kovesdy CP (2010). Kidney bone disease and mortality in CKD: revisiting the role of vitamin D, calcimimetics, alkaline phosphatase, and minerals. Kidney Int Suppl.

[10] Group KDIGOCKDMBDW (2009). KDIGO clinical practice guideline for the diagnosis, evaluation, prevention, and treatment of chronic kidney disease-mineral and bone disorder (CKD-MBD). Kidney Int Suppl.

[11] Shinaberger CS, Greenland S, Kopple JD, Van Wyck D, Mehrotra R, Kovesdy CP (2008). Is controlling phosphorus by decreasing dietary protein intake beneficial or harmful in persons with chronic kidney disease?. Am J Clin Nutr.

[12] Chiu Y-W, Teitelbaum I, Misra M, de Leon EM, Adzize T, Mehrotra R (2009). Pill burden, adherence, hyperphosphatemia, and quality of life in maintenance dialysis patients. Clin J Am Soc Nephrol.

[13] Chertow GM, Levin NW, Beck GJ, Depner TA, Eggers PW, Gassman JJ (2010). In-center hemodialysis six times per week versus three times per week. N Engl J Med.

[14] Culleton BF, Walsh M, Klarenbach SW, Mortis G, Scott-Douglas N, Quinn RR (2007). Effect of frequent nocturnal hemodialysis vs conventional hemodialysis on left ventricular mass and quality of life: a randomized controlled trial. JAMA..

[15] Rocco MV, Lockridge RS, Beck GJ, Eggers PW, Gassman JJ, Greene T (2011). The effects of frequent nocturnal home hemodialysis: the frequent hemodialysis network nocturnal trial. Kidney Int.

[16] Cornelis T, van der Sande FM, Eloot S, Cardinaels E, Bekers O, Damoiseaux J (2014). Acute hemodynamic response and uremic toxin removal in conventional and extended hemodialysis and hemodiafiltration: a randomized crossover study. Am J Kidney Dis.

[17] Meijers B, Toussaint ND, Meyer T, Bammens B, Verbeke K, Vanrenterghem Y (2011). Reduction in protein-bound solutes unacceptable as marker of dialysis efficacy during alternate-night nocturnal hemodialysis. Am J Nephrol.

[18] Tentori F, Zhang J, Li Y, Karaboyas A, Kerr P, Saran R (2012). Longer dialysis session length is associated with better intermediate outcomes and survival among patients on in-center three times per week hemodialysis: results from the Dialysis outcomes and practice patterns study (DOPPS). Nephrol Dial Transplant.

[19] Jardine MJ, Zuo L, Gray NA, JRd Z, Chan CT, Gallagher MP (2017). A Trial of Extending hemodialysis hours and quality of life. J Am Soc Nephrol.

[20] Jardine MJ, Zuo LI, Gray NA, de Zoysa J, Chan CT, Gallagher MP (2015). Design and participant baseline characteristics of 'A clinical trial of IntensiVE Dialysis': the ACTIVE Dialysis study. Nephrology..

[21] Vervloet MG, van Ballegooijen AJ (2018). Prevention and treatment of hyperphosphatemia in chronic kidney disease. Kidney Int.

[22] Isakova T, Gutierrez OM, Chang Y, Shah A, Tamez H, Smith K (2009). Phosphorus binders and survival on hemodialysis. J Am Soc Nephrol.

[23] Daugirdas JT, Chertow GM, Larive B, Pierratos A, Greene T, Ayus JC (2012). Effects of frequent hemodialysis on measures of CKD mineral and bone disorder. J Am Soc Nephrol.

[24] Laursen SH, Vestergaard P, Hejlesen OK (2018). Phosphate kinetic models in hemodialysis: a systematic review. Am J Kidney Dis.

[25] Fernandez-Martin JL, Martinez-Camblor P, Dionisi MP, Floege J, Ketteler M, London G (2015). Improvement of mineral and bone metabolism markers is associated with better survival in haemodialysis patients: the COSMOS study. Nephrol Dial Transplant.

[26] Piccoli Giorgina, Moio Maria, Fois Antioco, Sofronie Andreea, Gendrot Lurlinys, Cabiddu Gianfranca, D’Alessandro Claudia, Cupisti Adamasco (2017). The Diet and Haemodialysis Dyad: Three Eras, Four Open Questions and Four Paradoxes. A Narrative Review, Towards a Personalized, Patient-Centered Approach. Nutrients.

[27] Palmer SC, Hanson CS, Craig JC, Strippoli GF, Ruospo M, Campbell K (2015). Dietary and fluid restrictions in CKD: a thematic synthesis of patient views from qualitative studies. Am J Kidney Dis.

[28] Levine BS, Rodriguez M, Felsenfeld AJ (2014). Serum calcium and bone: effect of PTH, phosphate, vitamin D and uremia. Nefrologia..

[29] Bailie GR, Mason NA (2013). 2013: Dialysis of drugs.

