# Supplementary material for: Effect of extended hours dialysis on markers of chronic kidney disease-mineral and bone disorder in the ACTIVE Dialysis study
Source: BMC Nephrol. 2019 Jul 12;20:258. doi: 10.1186/s12882-019-1438-3 (PMC6624904; doi:10.1186/s12882-019-1438-3)

Supplementary Appendix

[Table S1: Additional baseline characteristics 2](#_Toc12040138)

[Table S2: Dialysate composition, flow rates and use of HDF over the duration of the study 3](#_Toc12040139)

[Table S3: Serum parameters and dialysis adequacy over the duration of the study 5](#_Toc12040140)

[Table S4: Blood pressure and fluid status changes over the duration of the study 7](#_Toc12040141)

[Figure S1: Dialysate calcium over the duration of the study 8](#_Toc12040142)

## **Table S1**: Additional baseline characteristics

| **Characteristics** | **Standard (n=100)** | **Extended (n=100)** | **Total (n=200)** | **P-value** |
| --- | --- | --- | --- | --- |
| Ideal Body Weight (kg) | 77.6 (19.3) | 73.9 (20.8) | 75.7 (20.1) | 0.19 |
| Height (cm) | 170 (8.5) | 170 (7.5) | 170 (8.0) | 0.66 |
| Cause of primary renal disease |  |  |  |  |
| Diabetic nephropathy | 34 (34.0) | 27 (27.0) | 61 (30.5) | 0.75 |
| Hypertension/Vascular disease | 11 (11.0) | 11 (11.0) | 22 (11.0) |  |
| Glomerulonephritis | 34 (34.0) | 41 (41.0) | 75 (37.5) |  |
| Polycystic kidney disease | 7 (7.0) | 5 (5.0) | 12 (6.0) |  |
| Other | 14 (14.0) | 16 (16.0) | 30 (15.0) |  |
| Co-morbidities |  |  |  |  |
| Diabetes | 39 (39.0) | 34 (34.0) | 73 (36.5) | 0.43 |
| Cardiovascular disease | 27 (27.0) | 22 (22.0) | 49 (24.5) | 0.41 |
| Congestive cardiac failure | 13 (13.0) | 19 (19.0) | 32 (16.0) | 0.25 |
| Dialysis access |  |  |  |  |
| Native arteriovenous fistula | 81 (81.0) | 87 (87.0) | 168 (84.0) | 0.70 |
| Synthetic arteriovenous fistula (graft) | 4 (4.0) | 3 (3.0) | 7 (3.5) |  |
| Tunnelled vascular catheter | 14 (14.0) | 9 (9.0) | 23 (11.5) |  |
| Other | 1 (1.0) | 1 (1.0) | 2 (1.0) |  |
| Membrane flux |  |  |  |  |
| High | 90 (90.0) | 82 (82.0) | 172 (86.0) | 0.24 |
| Low | 7 (7.0) | 11 (11.0) | 18 (9.0) |  |
| N/A | 3 (3.0) | 7 (7.0) | 10 (5.0) |  |
| Haemodiafiltration | 22 (22.0) | 20 (20.0) | 42 (21.0) | 0.73 |
| Blood flow rate (ml/min), median (IQR) | 280 (250–300) | 288 (250–300) | 288 (250–300) | 0.87 |
| Dialysate flow rate (ml/min), median (IQR) | 500 (500–500) | 500 (500–500) | 500 (500–500) | 0.26 |

## **Table S2**: Dialysate composition, flow rates and use of HDF over the duration of the study

|  | n/N | Standard | n/N | Extended | *P-value* |
| --- | --- | --- | --- | --- | --- |
| Sodium |  |  |  |  |  |
| Baseline | 100/100 | 140 (138–140) | 100/100 | 140 (138–140) | *0.64* |
| Month 3 | 96/98 | 140 (138–140) | 97/99 | 140 (138–140) | *0.91* |
| Month 6 | 95/96 | 140 (138–140) | 92/98 | 140 (138–140) | *0.73* |
| Month 9 | 93/94 | 140 (138–140) | 91/96 | 140 (138–140) | *0.49* |
| Month 12 | 90/92 | 140 (138–140) | 89/93 | 140 (138–140) | *0.48* |
| *P-trend* |  | *0.40* |  | *0.75* |  |
|  |  |  |  |  |  |
| Potassium |  |  |  |  |  |
| Baseline | 100/100 | 2.0 (2.0–2.5) | 100/100 | 2.0 (2.0–2.0) | *0.36* |
| Month 3 | 96/98 | 2.0 (2.0–2.5) | 97/99 | 2.0 (2.0–2.5) | *0.49* |
| Month 6 | 95/96 | 2.0 (2.0–2.5) | 92/98 | 2.0 (2.0–2.5) | *0.27* |
| Month 9 | 93/94 | 2.0 (2.0–2.5) | 91/96 | 2.0 (2.0–2.5) | *0.20* |
| Month 12 | 90/92 | 2.0 (2.0–2.5) | 89/93 | 2.0 (2.0–2.5) | *0.74* |
| *P-trend* |  | *0.69* |  | *0.057* |  |
|  |  |  |  |  |  |
| Calcium |  |  |  |  |  |
| Baseline | 100/100 | 1.5 (1.3–1.5) | 100/100 | 1.5 (1.3–1.5) | *0.75* |
| Month 3 | 96/98 | 1.5 (1.3–1.5) | 97/99 | 1.5 (1.3–1.5) | *0.62* |
| Month 6 | 95/96 | 1.5 (1.3–1.5) | 92/98 | 1.5 (1.3–1.5) | *0.45* |
| Month 9 | 93/94 | 1.5 (1.3–1.5) | 91/96 | 1.5 (1.3–1.5) | *0.71* |
| Month 12 | 90/92 | 1.5 (1.3–1.5) | 89/93 | 1.5 (1.3–1.5) | *0.91* |
| *P-trend* |  | *0.51* |  | *0.94* |  |
|  |  |  |  |  |  |
| Dialysate flow rate |  |  |  |  |  |
| Baseline | 100/100 | 500 (500–500) | 100/100 | 500 (500–500) | *0.26* |
| Month 3 | 96/98 | 500 (500–500) | 95/99 | 500 (500–500) | *0.0041* |
| Month 6 | 95/96 | 500 (500–500) | 90/98 | 500 (500–500) | *0.0041* |
| Month 9 | 93/94 | 500 (500–500) | 89/96 | 500 (500–500) | *0.076* |
| Month 12 | 89/92 | 500 (500–500) | 87/93 | 500 (500–500) | *0.58* |
| *P-trend* |  | *0.16* |  | *0.011* |  |
|  |  |  | *Mean difference*  *25ml/min (95%CI 9, 42)* | | *0.003^1^* |
|  |  |  |  |  |  |
| Blood flow rate |  |  |  |  |  |
| Baseline | 100/100 | 280 (250–300) | 100/100 | 288 (250–300) | *0.87* |
| Month 3 | 96/98 | 280 (250–300) | 95/99 | 260 (240–300) | *0.023* |
| Month 6 | 95/96 | 280 (250–300) | 90/98 | 250 (220–300) | *0.0007* |
| Month 9 | 93/94 | 280 (250–300) | 89/96 | 250 (230–300) | *0.0003* |
| Month 12 | 89/92 | 280 (250–300) | 87/93 | 250 (230–300) | *0.0007* |
| *P-trend* |  | *0.73* |  | *<0.001* |  |
|  |  |  | *Mean difference*  *23ml/min (95% CI 11, 34)* | | *<0.001^1^* |
|  |  |  |  |  |  |
| Proportion using HDF (%) |  |  |  |  |  |
| Baseline | 100/100 | 22.0 | 100/100 | 20.0 | *0.73* |
| Month 3 | 96/98 | 18.8 | 94/99 | 9.6 | *0.07* |
| Month 6 | 95/96 | 22.1 | 90/98 | 14.4 | *0.18* |
| Month 9 | 93/94 | 20.4 | 89/96 | 15.7 | *0.41* |
| Month 12 | 90/92 | 27.8 | 87/93 | 17.2 | *0.094* |
| *P-trend* |  |  | *OR for HDF after baseline*  *0.32 (95%CI 0.01, 1.02)* | | *0.056^2^* |
|  |  |  |  |  |  |

^1^ Mixed linear regression model of flow rate by treatment allocation over the four follow up visits, adjusted by time.

**^2^** Mixed logistic regression model of use of HDF by treatment allocation over the four follow up visits, adjusted by time.

## **Table S3**: Serum parameters and dialysis adequacy over the duration of the study

Mean (SD) or Median (IQR)

|  | n/N | Standard | n/N | Extended | *P-value* |
| --- | --- | --- | --- | --- | --- |
| Sodium |  |  |  |  |  |
| Baseline | 98/100 | 138.0 (3.3) | 98/100 | 138.6 (3.2) | *0.17* |
| Month 3 | 92/98 | 137.8 (3.1) | 93/99 | 138.3 (3.1) | *0.27* |
| Month 6 | 87/96 | 137.9 (3.3) | 87/98 | 138.0 (3.5) | *0.84* |
| Month 9 | 91/94 | 138.1 (3.0) | 86/96 | 138.0 (3.4) | *0.80* |
| Month 12 | 89/92 | 138.4 (3.3) | 85/93 | 138.7 (4.0) | *0.60* |
| *P-trend* |  | *0.48* |  | *0.33* |  |
|  |  |  |  |  |  |
| Potassium |  |  |  |  |  |
| Baseline | 99/100 | 4.97 (0.73) | 98/100 | 4.96 (0.76) | *0.87* |
| Month 3 | 93/98 | 4.99 (0.70) | 93/99 | 4.63 (0.70) | *0.0003* |
| Month 6 | 89/96 | 5.05 (0.79) | 88/98 | 4.69 (0.69) | *0.0016* |
| Month 9 | 91/94 | 4.99 (0.69) | 86/96 | 4.70 (0.82) | *0.0129* |
| Month 12 | 89/92 | 5.18 (0.76) | 86/93 | 4.71 (0.66) | *<0.0001* |
| *P-trend* |  | *0.07* |  | *0.0042* |  |
|  |  |  |  |  |  |
| Calcium |  |  |  |  |  |
| Baseline | 90/100 | 2.28 (0.24) | 88/100 | 2.27 (0.20) | *0.72* |
| Month 3 | 83/98 | 2.29 (0.20) | 80/99 | 2.31 (0.19) | *0.59* |
| Month 6 | 81/96 | 2.29 (0.24) | 74/98 | 2.30 (0.19) | *0.72* |
| Month 9 | 81/94 | 2.26 (0.22) | 70/96 | 2.35 (0.23) | *0.0165* |
| Month 12 | 76/92 | 2.26 (0.18) | 72/93 | 2.31 (0.22) | *0.22* |
| *P-trend* |  | *0.28* |  | *0.0459* |  |
|  |  |  |  |  |  |
| Phosphate |  |  |  |  |  |
| Baseline | 99/100 | 1.77 (0.59) | 98/100 | 1.86 (0.53) | *0.31* |
| Month 3 | 91/98 | 1.71 (0.52) | 91/99 | 1.51 (0.49) | *0.01* |
| Month 6 | 89/96 | 1.81 (0.50) | 88/98 | 1.52 (0.43) | *0.0001* |
| Month 9 | 89/94 | 1.73 (0.47) | 82/96 | 1.51 (0.45) | *0.0021* |
| Month 12 | 87/92 | 1.79 (0.50) | 85/93 | 1.55 (0.42) | *0.0005* |
| *P-trend* |  | *0.33* |  | *<0.0001* |  |
|  |  |  |  |  |  |
| PTH |  |  |  |  |  |
| Baseline | 87/100 | 20.7 (10.9–43.5) | 88/100 | 28.5 (11.7–46.2) | *0.33* |
| Month 3 | 80/98 | 20.2 (8.9–42.3) | 80/99 | 23.8 (9.1–53.8) | *0.53* |
| Month 6 | 75/96 | 21.0 (6.6–56.3) | 77/98 | 28.5 (13.0–51.9) | *0.13* |
| Month 9 | 75/94 | 27.2 (10.5–62.8) | 72/96 | 24.1 (6.9–50.6) | *0.54* |
| Month 12 | 72/92 | 18.6 (7.1–48.2) | 70/93 | 29.9 (11.4–55.0) | *0.15* |
| *P-trend* |  | *0.88* |  | *0.85* |  |
|  |  |  |  |  |  |
| Bicarbonate |  |  |  |  |  |
| Baseline | 91/100 | 22.8 (3.9) | 92/100 | 22.9 (3.5) | *0.90* |
| Month 3 | 88/98 | 23.1 (3.9) | 89/99 | 23.6 (4.0) | *0.42* |
| Month 6 | 80/96 | 22.8 (3.9) | 85/98 | 22.7 (3.8) | *0.77* |
| Month 9 | 82/94 | 22.8 (3.6) | 81/96 | 23.2 (3.5) | *0.55* |
| Month 12 | 87/92 | 22.4 (2.9) | 81/93 | 22.9 (3.5) | *0.31* |
| *P-trend* |  | *0.26* |  | *0.20* |  |
|  |  |  |  |  |  |
| Kt/V |  |  |  |  |  |
| Baseline | 63/100 | 1.42 (1.20–1.60) | 55/100 | 1.53 (1.26–1.80) | *0.12* |
| Month 3 | 60/98 | 1.44 (1.26–1.71) | 54/99 | 1.80 (1.53–2.21) | *<0.0001* |
| Month 6 | 56/96 | 1.46 (1.25–1.68) | 49/98 | 1.92 (1.53–2.34) | *<0.0001* |
| Month 9 | 53/94 | 1.39 (1.23–1.66) | 49/96 | 1.94 (1.52–2.19) | *<0.0001* |
| Month 12 | 55/92 | 1.40 (1.24–1.66) | 54/93 | 1.62 (1.44–2.30) | *0.0016* |
| *P-trend* |  | *0.99* |  | *0.051* |  |

## **Table S4**: Blood pressure and fluid status changes over the duration of the study

Mean (SD)

|  | n/N | Standard | n/N | Extended | *P-value* |
| --- | --- | --- | --- | --- | --- |
| Systolic BP (mmHg) | |  |  |  |  |
| Baseline | 100/100 | 138.8 (21.0) | 100/100 | 140.7 (20.7) | *0.52* |
| Month 3 | 96/98 | 139.4 (20.5) | 95/99 | 136.4 (21.4) | *0.36* |
| Month 6 | 94/96 | 140.7 (23.4) | 90/98 | 132.3 (20.5) | *0.01* |
| Month 9 | 93/94 | 134.2 (19.1) | 89/96 | 135.1 (19.4) | *0.75* |
| Month 12 | 91/92 | 133.8 (21.9) | 87/93 | 135.1 (22.3) | *0.70* |
| *P-trend* |  | *0.003* |  | *0.01* |  |
|  |  |  |  |  |  |
| Diastolic BP (mmHg) | |  |  |  |  |
| Baseline | 100/100 | 78.7 (13.6) | 100/100 | 80.6 (13.9) | *0.33* |
| Month 3 | 96/98 | 78.5 (12.2) | 95/99 | 76.5 (13.8) | *0.27* |
| Month 6 | 94/96 | 79.5 (12.9) | 90/98 | 77.1 (11.8) | *0.20* |
| Month 9 | 93/94 | 75.8 (11.0) | 89/96 | 76.4 (12.6) | *0.73* |
| Month 12 | 91/92 | 74.8 (10.9) | 87/93 | 76.1 (13.4) | *0.47* |
| *P-trend* |  | *0.001* |  | *0.004* |  |
|  |  |  |  |  |  |
| Interdialytic weight gain (kg) | | |  |  |  |
| Baseline | 99/100 | 2.1 (1.6) | 100/100 | 2.0 (1.9) | *0.63* |
| Month 3 | 96/98 | 2.3 (1.7) | 93/99 | 2.1 (2.0) | *0.55* |
| Month 6 | 94/96 | 2.4 (1.8) | 89/98 | 2.2 (1.5) | *0.53* |
| Month 9 | 92/94 | 2.5 (1.7) | 89/96 | 2.4 (1.5) | *0.76* |
| Month 12 | 89/92 | 2.4 (1.6) | 87/93 | 2.0 (1.5) | *0.14* |
| *P-trend* |  | *0.60* |  |  | *0.20* |
|  |  |  |  |  |  |

Figure S1: Dialysate calcium over the duration of the study (proportion of participants; mmol/l)


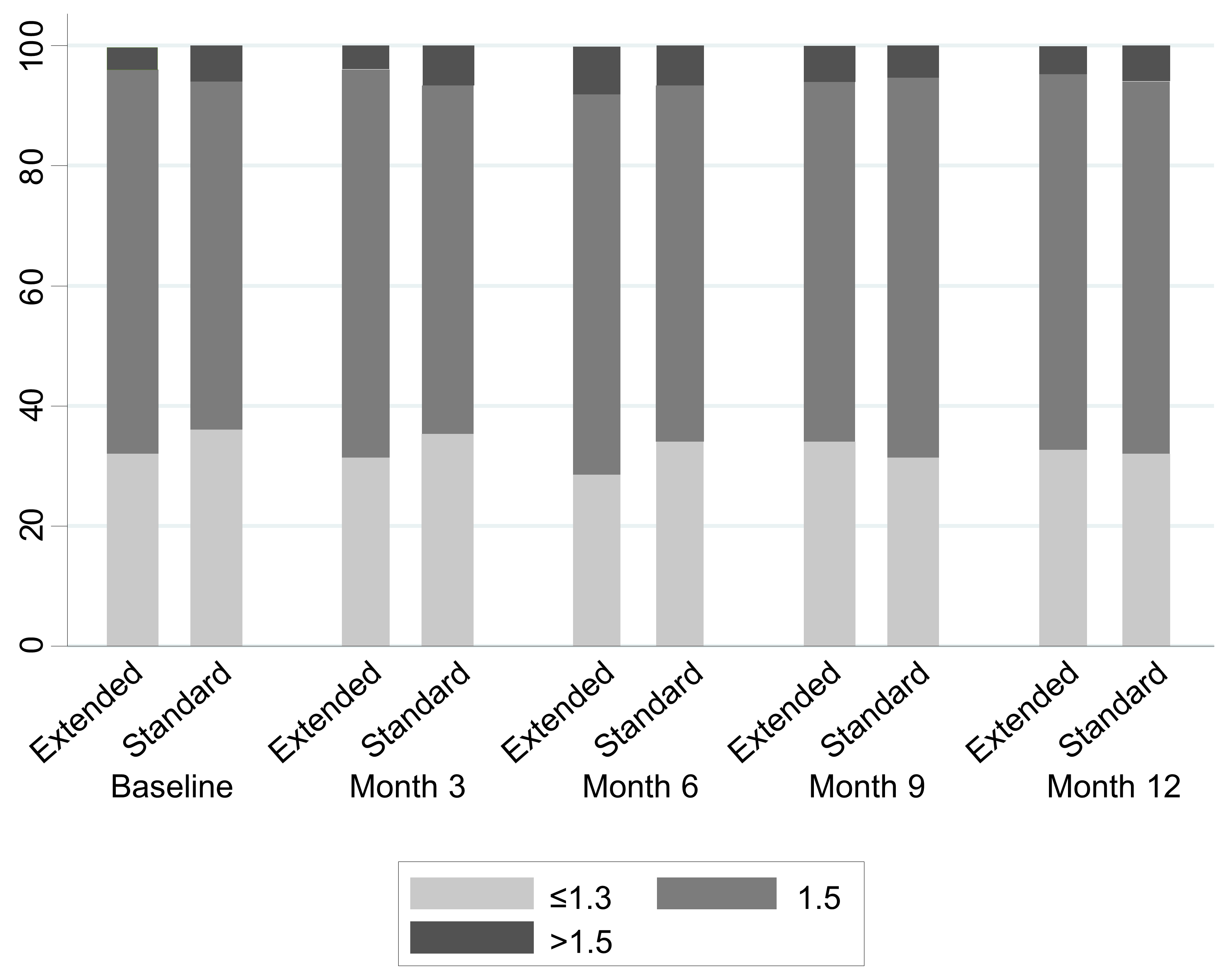

Supplement: Supplementary file 1 — : Table S1. Additional baseline characteristics. Table S2. Dialysate composition, flow rates and use of HDF over the duration of the study. Table S3. Serum parameters and dialysis adequacy over the duration of the study. Table S4. Blood pressure and fluid status changes over the duration of the study. Figure S1. Dialysate calcium over the duration of the study. (DOCX 264 kb) [file 12882_2019_1438_MOESM1_ESM.docx]
